# Does high COVID-19 spread impact neighbouring countries? Quasi-experimental evidence from the first year of the pandemic in Ireland

**DOI:** 10.12688/hrbopenres.13263.2

**Published:** 2021-09-06

**Authors:** Rakesh Ahmed, Peter May

**Affiliations:** 1 Centre for Health Policy and Management, Trinity College Dublin, Dublin, D2, Ireland; 2 The Irish Longitudinal Study on Ageing (TILDA), Trinity College Dublin, Dublin, D2, Ireland

**Keywords:** coronavirus, epidemiology, public health, policy, socioeconomic, population density, Ireland, instrumental variable

## Abstract

**Background *:*** Coronavirus disease 2019 (COVID-19) has necessitated public health responses on an unprecedented scale. Controlling infectious diseases requires understanding of the conditions that increase spread. Prior studies have identified sociodemographic, epidemiological and geographic associations. Ireland offers an unusual opportunity to quantify how high infection rates in one country impacted cases in a neighbouring country.

**Methods *:*** We analysed official statistics on confirmed COVID-19 cases on the island of Ireland for 52 weeks from March 2020. Our main research question was: Did higher cases in Northern Ireland (NI) impact the number of cases in the Republic of Ireland (ROI)? We used least squares regression to compare confirmed cases in ROI counties that border NI with the rest of the state. We included in our model sociodemographic, epidemiological and geographic factors. We employed the latitude of each county town as an instrumental variable to isolate a quasi-experimental estimate of the cross-border spread.

**Results *:*** In the quasi-experimental framework, and controlling for population density, age distribution and circulatory disease prevalence, border counties had an extra 21.0 (95%CI: 8.4-33.6) confirmed COVID-19 cases per 1000 people. This equates to an estimated 9,611 additional cases in ROI, or 4% of the national total in the first year of the pandemic. Our results were substantively similar in non-experimental frameworks, with alternative additional predictors, and in sensitivity analyses. Additionally, population density in ROI counties was positively associated with confirmed cases and higher proportions of residents in the professional classes was negatively associated.

**Conclusion *:*** On the island of Ireland during the first year of the COVID-19 pandemic, high infection rates in NI increased cases in the neighbouring ROI. Maximising co-ordination of pandemic responses among neighbouring countries is essential to minimising disease spread, and its associated disruptions to society and the economy. Socioeconomic disadvantage appeared to confer significant additional risk of spread.

## Introduction

### Background

Coronavirus disease 2019 (COVID-19) was the defining global event of 2020. The pandemic caused disruption to daily life in virtually every country in the world, necessitating public health responses on an unprecedented scale with consequences for population health; mortality, particularly among the oldest old; mental health; lifestyle and behavioural health; and the wider economy and society ^1^.

Controlling infectious diseases requires understanding of the conditions that increase spread, and multiple studies have examined the sociodemographic, epidemiological and geographic factors associated with infection rates in different countries ^2^. International comparative studies are recognised as essential for disease control ^3^, but these are challenging to conduct ^4^.

### Rationale

**Figure 1.**
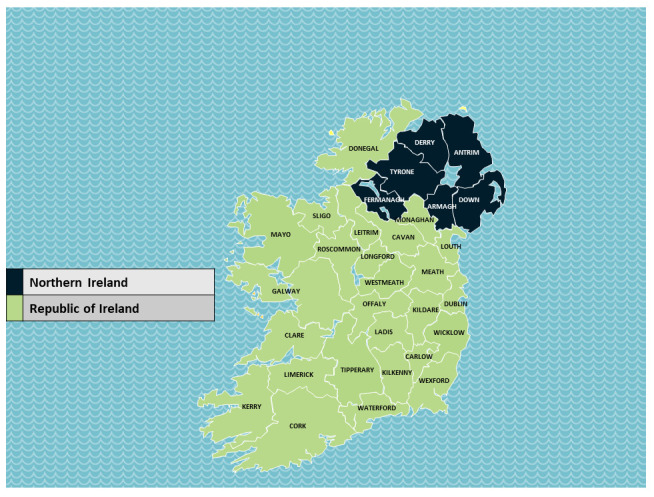
Map of Ireland. Map source: template adapted from Presentation Magazine ( www.presentationmagazine.com).

Ireland offers an unusual opportunity to isolate if high infection rates in one country significantly impacted infection rates in a neighbouring country. The island of Ireland comprises a single epidemiological unit covered by two legislative jurisdictions: the 26-county Republic of Ireland (ROI), which is an independent nation, and the six-county Northern Ireland (NI), which is a part of the United Kingdom. See Figure 1.

**Figure 2.**
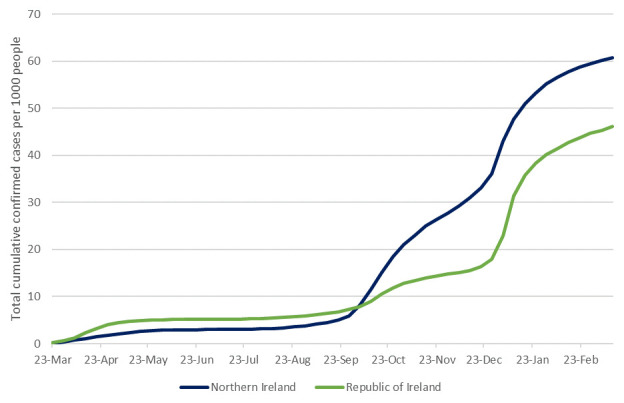
Total confirmed COVID-19 cases per 1000 people to 15th March 2021, in NI and ROI. Data sources: ^12, 13^.

The ROI has a centralised government in the capital city, Dublin. In NI, legislative responsibility rests with the Stormont Executive in Belfast, which has powers on health and a wide range of other policy matters devolved from the UK government in London. Each made decisions to control spread of the virus through 2020. The first cases of COVID-19 on either side of the border were confirmed in late February 2020 ^5^, and both governments initiated a wide-ranging lockdown of economic and societal activity to control this ‘first wave’ ^6, 7^. Restrictions were eased through the summer and then tightened again in anticipation of a ‘second wave’ of cases in the autumn ^6, 8^. ROI responded more quickly to rising cases in August and September, and experienced a relatively modest “second wave ^6^. In NI, Stormont officials prioritised co-ordination with London over Dublin and experienced among the highest per-capita rates in the world ^9^. Both jurisdictions eased “second wave” restrictions at the end of November and witnessed a very significant “third wave”, mostly driven by the B.1.1.7 variant, which first emerged in southeastern England in September and was transmitted to both jurisdictions at a high rate as people travelled home to Ireland for the Christmas holidays ^10, 11^. Total weekly cases per 1000 people, illustrating similar rates until the second wave and a much higher rate of confirmed cases in NI since, are presented in Figure 2 ^12, 13^.

### Aim

Our research question was:

Did higher COVID-19 numbers in NI impact the number of confirmed COVID-19 cases in the Republic of Ireland?

We addressed this question by investigating if the infection rate was different in those ROI counties that border NI, compared to the rest of ROI and while controlling for other factors related to socio-demography, epidemiology and geography. We hypothesised that numbers would be higher at the border, reflecting differing public health responses in Belfast and Dublin, and the overspill of policy effects from NI into ROI border counties. This study is reported according to the STROBE (Strengthening the Reporting of Observational Studies in Epidemiology) guidelines ^14^.

## Methods

### Study design

We analysed the number of confirmed COVID-19 cases in ROI for the period March 2020 to March 2021 inclusive. We created a dataset at the county level, i.e. with 26 series, using official, publicly available statistics ^15^.

Our outcome of interest was total number of confirmed cases per 1000 people, over 52 weeks from the start of official statistics reporting in March 2020. Since the whole of the ROI was under the same testing and reporting system, outcome data should be highly comparable across counties.

Our primary predictor of interest was binary: does the ROI county border NI? If high COVID-19 rates in NI did impact ROI, then the border counties are the most likely place to observe the overspill. There have been no travel restrictions between ROI and NI since the ‘Good Friday Agreement’ in 1998, and many people live, work, shop and socialise on both sides of the border. There is no third jurisdiction on the island as a potential confounding factor. In terms of epidemiology and human geography, Ireland’s population is homogenous by the standards of other high-income European countries and the two jurisdictions are often considered a single epidemiological unit, e.g. sharing an all-island institute for public health ^16^. Given this consistency of data reporting, homogeneity of population and small country size, we consider that differential rates in border counties are most plausibly explained by proximity to NI.

To check our main results for possible unobserved confounding, we also employed a quasi-experimental framework. A quasi-experiment infers the causal effect of an exposure on an outcome when that exposure cannot be randomly assigned ^17^. One such quasi-experimental method is an instrumental variable approach. An instrumental variable is a factor that is associated with the exposure of interest but neither the outcome of interest (except via the exposure) nor any other predictor in the model ^18^. Geographical factors are commonly used as instruments in environmental sciences ^19^ and economics ^20^; and are additionally important in using region-level data like ours due to the risk of ecological fallacy ^21^. We employed geographic latitude as an instrument, since among ROI counties northern-ness is by definition associated with proximity to the NI border but it has no plausible association with other factors in our model.

We took additional predictors on the socio-demographics, epidemiology and geography of each county from the government Central Statistics Office (CSO), which also ensures high consistency of data among independent variables.

### Setting and context

Ireland is an island in north-western Europe. It is split into 32 counties, 26 of which make up the ROI with the remaining six making up the UK nation of NI. Contextual differences include population, which is approximately 4.7million in ROI and 1.9million in NI, and population density, which approximately 68 persons/km ^2^ in ROI and 137 persons/km ^2^ in NI ^22, 23^. The ROI healthcare system is a mixed public and private system, and in NI healthcare is generally provided for free, publicly by the National Health Service ^24^.

In January in the ROI, the National Public Health Emergency Team was created to manage the national response to the pandemic ^6^. They provided advice to the government on public health policy by assessing the situation through the country and conferring with the World Health Organisation and the European Centre for Disease Control and Prevention ^25^. Their remit included the aim, “To collaborate with colleagues in Northern Ireland in support of the agreed programme of co-operation and collaboration” ^25^. In NI, the Department of Health and Public Health Agency work with partners in the UK and the Irish system to produce public health regulations during this time ^26^.

### Variables and sources

***Dependent variable.*** All data were at the ROI county level, i.e. we had 26 series. We extracted data weekly at 52 points, starting at the point that official statistics were reported in a standardised form, week of March 23 ^rd^, 2020. Therefore, our data cover 52 weeks of reporting, but the first official report reflected all cases to that point and the first case was identified at the end of February. We therefore have 52 time series points covering 55 weeks of cases.

Our main outcome variable was total number of confirmed COVID-19 cases per capita in each county in the study period. Daily confirmed cases in the ROI were reported on the day the person has been notified of a positive test result ^15^. A confirmed case was defined as detections of severe acute respiratory syndrome coronavirus 2 (SARS-CoV-2) nucleic acid in clinical specimen. The statutory Health Service Executive (HSE) made testing freely available for those who develop symptoms such as a cough, fever, or loss of taste or smell. Close contacts of a positive case also qualified for testing.

***Independent variables.*** Our main predictor of interest was binary: does the county border NI? Five counties had a value of 1 (Donegal, Leitrim, Cavan, Monaghan, Louth) and the other 21 counties a value of 0.

The CSO reports data by county on local characteristics that may also be associated with number of COVID-19 cases ^27^. We identified the following potentially useful county-level predictors:

- Age profile (since older people are more likely to show symptoms and therefore to be tested) ^2^,
- Female population (possible gender differences in susceptibility to the disease, and also in patterns of health care use including presentation for testing) ^2, 28^,
- Nursing home bed capacity (as nursing home residents more likely to show symptoms and are tested more often) ^29^,
- Percentage of 2018 deaths with primary cause respiratory disease (as this may be somewhat protective of COVID-19) ^30^,
- Percentage of 2018 deaths with primary cause circulatory disease (as existing circulatory disease is associated with severity of symptoms) ^2^,
- Social class (since those with high socioeconomic disadvantage appear at higher risk of catching the virus) ^31^,
- People living alone (those living alone do not have cohabitants to spread disease to and may complete a true self isolation at home if required),
- Population density (since spread will be faster among those living together more closely) ^32^,
- Region of the country (to control for other geographical factors, e.g. those counties that have average population density but border a large urban area) ^32^.

### Statistical methods and bias

We used two statistical approaches to assess our hypotheses: ordinary least squares (OLS) regression, and two-stage least squares regression (2SLS) with an instrumental variable. We chose OLS since this is a powerful, universally understood approach to statistical analysis of quantitative data and in our opinion the best way to understand association between all of our available predictors and outcome. The biggest weakness of OLS in our context is that there is no mechanism to control for confounding – that is, we might find a significant association between our primary predictor (border county) and our outcome (confirmed COVID cases per capita), but this result could not be considered causal. The observed difference might instead be explained by a third unobserved factor associated with both predictor and outcome.

To address this potential limitation we employed a quasi-experimental framework. We estimated a causal association between our primary predictor and outcome using 2SLS and an instrumental variable ^18^. Each county in Ireland has a ‘county town’ – typically the largest urban centre in the county and the location of local authority buildings. We used Google to get the latitude of each county town and instrumented this on our primary predictor. We tested the strength of the instrument using the F statistic and the rule of thumb that 10<F is acceptable ^33^.

For both OLS and 2SLS we built three models, where models were differentiated by choice of predictors. First, we evaluated the difference between per capita COVID-19 rates in border counties and elsewhere without adjusting for other factors. We label this the ‘basic model’. Second, we evaluated the difference controlling for those predictors suggested by information criteria prior to inspecting results ^34^. Only variables that reduced the Bayesian Information Criterion (BIC) were retained. We label this the ‘parsimonious model’. Third, we evaluated the difference in a hypothesis-driven model, controlling for all predictors identified as potentially useful. We label this the ‘hypothesis-driven model’. We assessed model performance on collinearity (variance inflation factor (VIF)), information loss (BIC), and goodness of fit (R ^2^), and compared the results.

We conducted two sensitivity analyses: adding NI as a 27 ^th^ series in the model, and recasting the outcome model as number of cases per capita aged over 65 (and thus explicitly controlling for ageing in the outcome, not in the predictors).

All analyses were performed in Stata (version 15) ^35^. Raw data and Stata code are provided as underlying data to ensure transparency and replicability. Due to the study design, there were no missing data.

## Results

### Descriptive data

County-level data, grouped by region, reporting total population and all available predictors, are summarised in Table 1 ^14^. For each predictor, variables in the top quartile are highlighted dark green and the bottom quartile are highlighted light green with the intermediate values in white. The five border counties comprising our exposure group do not exhibit any systematic differences from the other 21: of eight predictors evaluated for inclusion in the model (all variables in Table 1, excluding total population), the border counties have at least one county in both the highest and the lowest quartiles for six variables. Other regions show distinct characteristics. Notably, the Mid-East, incorporating Dublin and three bordering counties, accounts for 40% of national population, and this population is distinctively densely grouped as well as more heavily drawn from the professional, managerial and technical classes.

**Table 1.** County regions, populations and independent predictors.

County	Population *	Population density *	Gender balance *	Population ageing *	Live alone *	Social class *	Respiratory deaths +	Circulatory deaths +	NH bed per cap #
***Border***									
Cavan	76176	39.4	49.7%	13.7%	28.5%	29.4%	13.0%	28.0%	49.6
Donegal	159192	32.7	50.4%	15.1%	28.4%	29.9%	12.0%	32.0%	40.3
Leitrim	32044	20.2	49.9%	16.9%	32.7%	33.6%	14.0%	41.0%	47.5
Monaghan	61386	47.4	49.7%	14.0%	27.4%	29.3%	12.0%	30.0%	49.0
Louth	128884	156.0	50.6%	12.5%	26.4%	31.1%	12.0%	25.0%	39.1
***Midlands***									
Laois	84697	49.2	49.5%	11.3%	26.3%	32.2%	16.0%	25.0%	37.5
Longford	40873	37.5	49.6%	14.2%	29.5%	27.7%	16.0%	28.0%	50.0
Offaly	77961	39.0	50.2%	13.6%	24.5%	29.9%	14.0%	29.0%	50.0
Westmeath	88770	48.2	50.3%	12.8%	26.3%	33.6%	14.0%	29.0%	54.4
***West***									
Galway	258058	42.0	50.5%	13.5%	26.9%	36.8%	13.0%	29.0%	54.6
Mayo	130507	23.4	50.2%	17.6%	28.8%	31.7%	13.0%	32.0%	48.1
Roscommon	64544	25.3	49.8%	16.6%	29.5%	33.1%	14.0%	31.0%	67.1
Sligo	65535	35.7	50.6%	16.2%	28.6%	34.6%	12.0%	28.0%	36.5
*Mid-East*									
Dublin	1347359	1461.3	51.1%	12.2%	26.8%	40.5%	13.0%	28.0%	49.6
Kildare	222504	131.3	50.3%	9.9%	22.4%	37.9%	13.0%	28.0%	74.5
Meath	195044	83.3	50.4%	10.7%	22.7%	38.1%	16.0%	25.0%	52.0
Wicklow	142425	70.3	50.7%	13.0%	23.2%	40.4%	13.0%	26.0%	59.4
***Mid-West***									
Clare	118817	34.4	50.5%	14.9%	27.8%	35.9%	16.0%	28.0%	53.5
Limerick	194899	70.7	50.1%	14.1%	26.9%	33.6%	14.0%	27.0%	47.4
Tipperary	159553	37.1	50.1%	15.3%	27.7%	31.8%	13.0%	30.0%	48.0
***South-East***									
Carlow	56932	63.5	50.0%	12.9%	26.6%	30.2%	10.0%	34.0%	50.8
Kilkenny	99232	47.9	50.1%	14.2%	25.0%	37.2%	14.0%	30.0%	47.5
Waterford	116176	62.6	50.4%	15.0%	27.2%	32.9%	14.0%	29.0%	42.9
Wexford	149722	63.3	50.8%	14.7%	26.2%	30.9%	13.0%	33.0%	47.1
***South-West***									
Cork	542868	72.4	50.5%	13.6%	26.8%	36.9%	11.0%	31.0%	51.2
Kerry	147707	30.7	50.5%	16.9%	27.9%	31.6%	12.0%	32.0%	41.3

Sources: * 2016 Census ^22^, + CSO Births, Deaths and Marriages ^36^, # Health Information and Quality Authority ^37^ Colours: For each descriptive variable, top quartile results in dark green , bottom quartile results in light green . Legend:**Population:** Total number of residents; **Population density:** population/km ^2^; **Gender balance:** % of population=female; **Population ageing:** % of population aged 65+; **Live alone:** % of population aged 65+ who live alone; **Social class: %** of population aged<=65 in professional, managerial or technical classes; **Respiratory deaths:** proportion of deaths in 2018 with death certificate ICD-10 code primary cause J00-J99 ^38^; **Circulatory deaths:** proportion of deaths in 2018 with death certificate ICD-10 code primary cause I00-I99 ^38^; **NH bed per cap:** total nursing home beds/population.

### Outcome data

**Figure 3.**
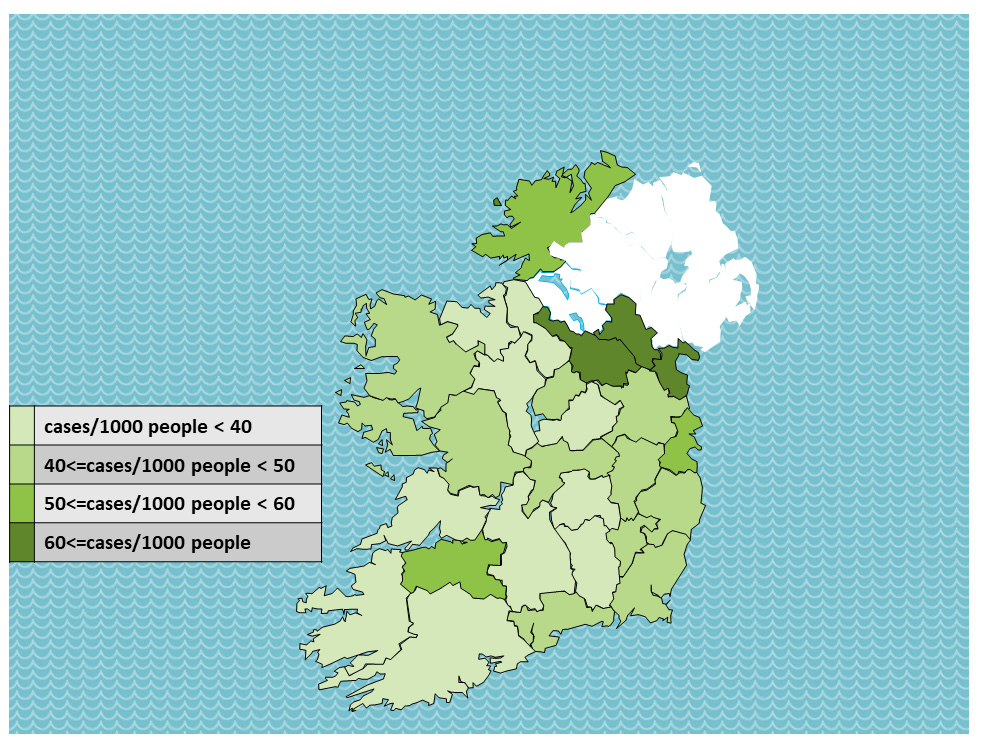
Total COVID-19 cases per 1000 people to 15th March 2021, by ROI county. Data source: ^12^ Map source: template adapted from Presentation Magazine ( www.presentationmagazine.com)

Total case numbers during the first year of the pandemic by region, and adjusting for total population, are presented in Table 2. Total case numbers per 1000 people are presented by county in Figure 3.

**Table 2.** Total case numbers and cases per capita in ROI, by region.

Region	Total number of cases	Cases/1000 people
Border	27633	60.4
Midland	11877	40.6
West	20167	38.9
Mid-East	79402	41.6
Mid-West	20612	43.6
South-East	18175	43.1
South-West	25862	37.4
**National**	**227790**	**47.8**

Source:^12^Legend: For county regions, see Table 1.

An apparent association between proximity to the border is observed. There were 227,790 total confirmed cases in the state, 47.8 per 1000 people. Of seven regions, only the border region (60.4 cases per 1000 people) was above the national mean.

At the county level, three counties had more than 60 cases per 1000 people, and all three – Louth, Monaghan and Cavan – are at the border. Three more counties had more than 50 cases per 1000 people, and these were Donegal (border county), and Dublin and Limerick (major urban centres). The fifth border county, Leitrim, did not have high case numbers.

### OLS output

Output for the OLS regressions is provided in Table 3. With respect to diagnostics, different models performed better on different measures. The basic model by definition performed best on collinearity. The parsimonious model included two additional predictors based on BIC – population density and historical level of circulatory disease – and performed best on information loss. The hypothesis-driven model performed best on R ^2^.

**Table 3.** OLS Regression Output.

	*Basic model:* *Border predictor* * only*		*Parsimonious model: * *predictors based * *on IC*		*Hypothesis-driven model: * *all available predictors*	
*Diagnostics*						
*Mean VIF*	*-*		*1.7*		*3.0*	
*BIC*	*204*		*196*		*199*	
*R ^2^*	*0.25*		*0.56*		*0.77*	
***Results***	*Coeff.*	*95% CI*	*Coeff.*	*95% CI*	*Coeff.*	*95% CI*
*Border county*	**15.7**	**4.3-27.2**	**19.5**	**10.0 to 29.0**	**16.7**	**6.4 to 26.9**
*CV deaths*			-120.9	-243.5 to 1.7	-123.9	-267.4 to 19.7
*Pop density*			**5.5**	**0.7 to 10.4**	**11.2**	**4.0 to 18.4**
*Pop ageing*					229.5	-186.5 to 645.5
*Female*					-545.0	-1963.7 to 873.7
*NH beds*					0.2	-0.3 to 0.7
*Respiratory deaths*					-70.1	-344.0 to 203.8
*Live alone*					-193.0	-519.0 to 133.1
*Social class*					**-183.8**	**-327.6 to -40.1**

Legend:**VIF:** variance inflation factor, a measure of collinearity where lower is better. **BIC:** Bayesian information criterion, a measure of information loss where lower is better. **R ^2^:** a measure of goodness of fit, where higher is better. **Coeff.:** coefficient. The estimated change in outcome with a one unit increase in the predictor, holding all other factors in the model constant. **CI:** confidence interval. **Border county**=1 if county borders Northern Ireland. **All other predictors:** see Table 1. Statistically significant (p<0.05) results highlighted bold.

In the basic model, the estimated association between our primary predictor – does the county border NI? – and our outcome – total COVID-19 cases in the first year of the pandemic, per 1000 people - was 15.7 (95% confidence interval (CI): 4.3-27.2; p=0.009). In the parsimonious model, the equivalent estimate was 19.5 (CI: 10.0-29.0; p<0.001), and in the hypothesis-driven model it was 16.7 (CI: 6.4-26.9; p=0.003). The other statistically significant associations between predictor and outcome were population density (positive association) and proportion of people in the professional, managerial and technical classes (negative association).

### 2SLS output

Output for the 2SLS regressions is provided in Table 4. The instrument performed satisfactorily (10<F) in the basic and parsimonious models but was weaker in the full model.

**Table 4.** 2SLS Regression Output.

	*Basic model:* * Border * *predictor only*		*Parsimonious * *model: predictors* * based on IC*		*Hypothesis-driven * *model: all available* * predictors*	
*Diagnostics*						
*F statistic*	*16*		*17*		*8*	
***Results***	*Coeff.*	*95% CI*	*Coeff.*	*95% CI*	*Coeff.*	*95% CI*
*Border county*	**18.5**	**1.9-35.0**	**21.0**	**8.4 to 33.6**	**20.9**	**8.0 to 33.9**

Legend:**F statistic:** a measure of instrument strength, where 10<F is considered acceptable. **Coeff.:** coefficient. The estimated change in outcome with a one unit increase in the predictor, holding all other factors in the model constant. **CI:** confidence interval. Statistically significant (p<0.05) results highlighted bold.

The estimated association between border county and outcome in these models are: 18.5 (CI: 1.9-35.0; p=0.03), 21.0 (CI: 8.4-33.6; p=0.001) and 20.9 (CI 8.0-33.9; p=0.002).

### Sensitivity analyses

We conducted two sensitivity analyses ^14^ adding NI as a 27 ^th^ series in the model, and recasting the outcome model as number of cases per capita aged over 65 (and thus explicitly controlling for ageing in the outcome, not in the predictors). Our results were substantively unaffected.

## Discussion

### Key findings

Ireland offers an unusual opportunity to investigate the movement of COVID-19 across international borders. Our analysis shows that on the island of Ireland for the first year of the pandemic, NI had more cases per capita than ROI, driven mainly by the “second wave” of infections in autumn 2020 ( Figure 2). Descriptive data show that among ROI counties, per capita cases where higher at the border than elsewhere ( Table 2, Figure 3).

In regression analyses, a significant association between border counties and the number of per capita cases is consistently observed ( Table 3). In a quasi-experimental framework, this relationship appears causal ( Table 4). The fundamental conclusion is robust to chosen predictors, and the instrumental variable results affirm the OLS output. All six results reported across Table 3 and Table 4 are substantively similar and in line with our prior hypothesis: they estimate that border counties had between 15.7 and 21.0 additional cases per 1000 people in the first year of the pandemic.

Other factors associated with confirmed cases were population density and social class, consistent with results that have been found in other countries.

### Limitations of this study

Our study has three main limitations. First, data are observational, and it is possible that a third unobserved factor associated with both COVID-19 spread and proximity to the Irish border has caused our results. We used an instrumental variable approach to minimise this concern as well as multiple sensitivity analyses. In the context of Ireland’s geography and society – an island with a single epidemiological unit and no differential restrictions on travel to Great Britain and the rest Europe - we consider this risk to be low. The ROI government took a small number of differential steps within the country during the pandemic, notably localised lockdowns in Dublin and border counties in October 2020 ^39^, but if affecting our results they bias them downwards. The second limitation is scope. It is possible that overspill of NI cases into ROI went beyond the border counties, but we were unable to test that using our framework. It is therefore likely that our estimates undercount the total number of cases in ROI arising from high spread in NI. Third, we do not isolate a specific policy choice that precipitated NI’s damaging second wave. Overall, the five British and Irish jurisdictions took similar measures but at slightly differing times, and ROI implemented restrictions earlier than NI prior to the second wave. Additionally, we acknowledge some repetition in our results reporting. In the final analysis neither model approach nor covariate choice nor instrument strength has any substantive impact on results and interpretation ^40^. However, we were not to know that at the start of the analysis and by reporting the results of different approaches we demonstrate robustness to these different factors. The strengths of our study are consistency of data and measurement, the unusual opportunity provided by Ireland to isolate the question of cross-border spread, and the opportunity to check our results in a quasi-experimental framework.

### Interpretation

At the most recent census, the five ROI border counties had a population of 457,682 ^22^. Therefore, our results suggests that, depending on the estimate chosen inferior COVID-19 control in NI led to between 7,186 and 9,611 cases in those counties. This is equivalent to 26–35% of cases in the five border counties, and 3–4% of cases in ROI nationally in the first year of the pandemic. The total number of deaths associated with this increased infection rate can only be known in time, disentangling those caused by and associated with COVID-19.

The corollary of our results is that neighbouring countries should maximise co-ordination of response. To minimise disease spread and its adverse events, co-ordination between jurisdictions that share a border is more important than co-ordination between jurisdictions that share a capital city. Multiple public figures in Ireland have suggested that high case rates at the border require an all-island response to infectious disease crises ^41– 43^. Our results provide strong scientific evidence of the problem, but the politics of the island preclude the obvious solution. Perhaps most important, our analysis quantifies only the number of COVID-19 cases in one jurisdiction due to high spread in another when both jurisdictions were practising uncoordinated cyclical lockdowns and easing of restrictions. True co-ordination across the island would extend beyond this model to a unified policy on international travel, whose estimated effect on COVID-19 cases would be much larger, but which is widely considered a political impossibility.

Our results complement and extend prior research on factors associated with COVID-19 spread ^2, 7, 28, 31, 32, 44– 45^. In particular, they complement findings elsewhere on the importance of regional and international co-ordination ^46– 49^. Associations between population density and social class, and the number of COVID-19 cases, are consistent with those results elsewhere. Socioeconomic disadvantage appeared to confer significant additional risk to infection, but this association was not identified as part of our causal analysis and requires further investigation ^21^.

## Conclusion

On the island of Ireland during the COVID-19 pandemic, high infection rates in NI increased cases in the neighbouring ROI. While some studies have examined factors associated with spread within countries, we are not aware of any prior study applying a causal framework to show how heightened rates in one country may impact another. Maximising co-ordination of pandemic responses among neighbouring countries is essential to minimising disease spread, and its associated disruptions to society and the economy. Such co-ordination would require politics deferring to science.

## Data availability

### Underlying data

Open Science Framework: Appendix to: [Does high Coronavirus-19 spread impact neighbouring countries? Evidence from Ireland]. 10.17605/OSF.IO/JKHFY ^14^.

This project contains the following underlying data:

- 20210317 Analytic dataset.xlsx – contains all data used in this study
- 20210317 IRLC19 submitted.do – Stata code to reproduce the analyses and results reported here, using ‘Analytic dataset’
- 20210317 IRLC19 submitted.smcl – Stata log of the analyses and results reported here

### Reporting guidelines

Open Science Framework: STROBE checklist for Does high COVID-19 spread impact neighbouring countries? Evidence from Ireland. https://doi.org/10.17605/OSF.IO/JKHFY ^14^.

Data are available under the terms of the Creative Commons Zero "No rights reserved" data waiver (CC0 1.0 Public domain dedication).
