# Supplementary material for: Does high COVID-19 spread impact neighbouring countries? Quasi-experimental evidence from the first year of the pandemic in Ireland
Source: HRB Open Res. 2021 Sep 6;4:56. Originally published 2021 May 20. [Version 2] doi: 10.12688/hrbopenres.13263.2 (PMC8287536; doi:10.12688/hrbopenres.13263.2)
Supplement: Peer review history

# Peer review: Reviewer response for version 1

**DOI:** 10.21956/hrbopenres.14435.r29588

**Published:** 2021-07-16

**Authors:** Neil Pearce

**Affiliations:** 1 Department of Medical Statistics, London School of Hygiene and Tropical Medicine, London, UK

This is an interesting and useful paper, and I think that the methods and conclusions are generally sound.

 My main questions are about the analyses, in particular the 'parsimonious model'. There is a large literature on why we don't need to have parsimonious models, and that we should not use p-values, goodness-of-fit, etc to decide on the final statistical model (see, e.g. Greenland S, Daniel R, Pearce N. Outcome modeling strategies in epidemiology: traditional methods and basic alternatives. International Journal of Epidemiology 2016; 45: 565-575.). ^1^ So the parsimonious model is a bit of a distraction, as well as not being ideal. On the other hand, the findings in all three models are similar.

 I also don't see the point of having the two different approaches shown in tables 3 and 4. If you want to keep these, I would have one table (ideally the format of table 3, with the basic model and the hypothesis-driven model), and move the rest to appendices

Is the work clearly and accurately presented and does it cite the current literature?

Yes

If applicable, is the statistical analysis and its interpretation appropriate?

Partly

Are all the source data underlying the results available to ensure full reproducibility?

Yes

Is the study design appropriate and is the work technically sound?

Yes

Are the conclusions drawn adequately supported by the results?

Yes

Are sufficient details of methods and analysis provided to allow replication by others?

Yes

Reviewer Expertise:

Epidemiological methods

I confirm that I have read this submission and believe that I have an appropriate level of expertise to confirm that it is of an acceptable scientific standard.

## References

[1] : Outcome modelling strategies in epidemiology: traditional methods and basic alternatives.*Int J Epidemiol*.45(2) : 10.1093/ije/dyw040565-7510.1093/ije/dyw04027097747PMC4864881

# Peer review: Reviewer response for version 1

**DOI:** 10.21956/hrbopenres.14435.r29508

**Published:** 2021-07-06

**Authors:** Aileen Murphy

**Affiliations:** 1 Department of Economics, Cork University Business School, University College Cork, Cork, Ireland

The authors analyse official statistics on confirmed COVID-19 cases for 52 weeks and examine if higher cases in NI impact the number of cases in ROI. Using least squares regression authors compared confirmed cases in ROI counties that border NI and control for socio-demographic, epidemiological and geographic factors. The results confirm border counties had additional COVID-19 cases per 1000. These results were consistent across the range of models /frameworks employed. Authors interpretation of the results presents a very interesting and relevant policy position – calling for co-ordinated responses among neighbour countries.

  

- Is the work clearly and accurately presented and does it cite the current literature?

The analysis is very clearly and accurately presented. Current national literature is cited in initial set up and grey literature (opinion pieces / newspaper articles) are heavily relied upon in the discussion. While the latter are useful for context more international sources (and academic were feasible) would be useful as while the ROI / NI border represents an opportunity to investigate the phenomenon of high infection rates in one country significantly impacting infection rates in a neighbouring country the situation and learnings are not entirely unique to ROI/NI, there are other examples across Europe and internationally. 

 Note wrong table numbers occasionally referred to in the text e.g. Page 6 the 2LS is on Table 4 not Table 3.

 On Table 3 – the legend should include that coefficients in bold are statistically significant.

 Page numbers on the STROBE statement do not correspond with PDF version of submitted manuscript.

  

- Is the study design appropriate and does the work have academic merit?

Authors constructed a data set using official statistics and the study design employed to analyse the data is appropriate. The authors clearly outline the methods, which are appropriate.

  

- Are sufficient details of methods and analysis provided to allow replication by others?

Presentation of the independent variables with reference to literature on page 5 is transparent. Authors clearly present the methods and analyses techniques to facility replication. The stata code and raw data are available.

  

- If applicable, is the statistical analysis and its interpretation appropriate?

The statistical analysis and its interpretation are appropriate. However, as the authors adopt two methodological approaches – OLS (Table 3) and 2LS (Table 4) a note outlining the motivation for performing both techniques would be beneficial. 

 Also consistency between terminology used would be desirable between description of methods and the discussion. For a non-expert audience moving from LS and 2LS to multivariate regression analysis and quasi-experimental framework could be confusing.

  

- Are all the source data underlying the results available to ensure full reproducibility?

Yes, the raw data and stata code are available. The aforementioned data is clearly presented on Table 1.

- Are the conclusions drawn adequately supported by the results?

Authors do draw appropriate and adequately supported conclusions. Limitations of the analysis are clearly presented. Conclusions are well position in a timely policy context. As outlined above authors could refer to the transferability of results beyond the island of Ireland and refer to ongoing international discussions re same.

Is the work clearly and accurately presented and does it cite the current literature?

Yes

If applicable, is the statistical analysis and its interpretation appropriate?

Yes

Are all the source data underlying the results available to ensure full reproducibility?

Yes

Is the study design appropriate and is the work technically sound?

Yes

Are the conclusions drawn adequately supported by the results?

Yes

Are sufficient details of methods and analysis provided to allow replication by others?

Yes

Reviewer Expertise:

Health Economics

I confirm that I have read this submission and believe that I have an appropriate level of expertise to confirm that it is of an acceptable scientific standard.
